# Sorely Mistaken—Soft Palatal Myxedema in Decompensated Hypothyroidism Presenting as a Sore Throat: Case Report

**DOI:** 10.5811/cpcem.53121

**Published:** 2026-04-29

**Authors:** Gabriella Miller, Bennett Myers

**Affiliations:** University of Maryland School of Medicine, Department of Emergency Medicine, Baltimore, Maryland

**Keywords:** airway, endocrinology, myxedema, thyroid, case report

## Abstract

**Introduction:**

Oropharyngeal myxedema is a rare presenting symptom of decompensated hypothyroidism that can mimic more common causes of sore throat.

**Case Report:**

We describe a case of an older woman who presented with throat pain and dysphagia, found to have soft palate edema on exam and imaging. Laboratory testing confirmed severe hypothyroidism, and her symptoms eventually resolved with thyroid hormone replacement therapy.

**Conclusion:**

This case highlights a rare and under-recognized presentation of a common endocrine disorder. Consider myxedema from severe hypothyroidism in patients with subacute oropharyngeal pain and swelling. Without early recognition and treatment, the patient is at risk for two life-threatening conditions: airway compromise from soft palate myxedema and progression of hypothyroidism to myxedema coma.

## INTRODUCTION

Hypothyroidism can be a challenging diagnosis to make from the emergency department (ED) because of nonspecific presenting symptoms.^1^ Subacute oropharyngeal myxedema is an unusual presenting symptom of decompensated hypothyroidism and may be incorrectly attributed to alternate pathology such as a mass lesion, infection, or angioedema. It can lead to life-threatening airway compromise if early intervention is not achieved.^2^–^6^ We present a case of soft palatal myxedema diagnosed on direct visualization and confirmed by fiberoptic laryngoscopy, computed tomography (CT), and thyroid function laboratory testing. In consultation with an endocrinologist the patient was discharged with resolution of symptoms on short-term outpatient follow-up underscoring the importance of early intervention.

## CASE REPORT

A 68-year-old woman with a past medical history of Graves disease status-post radioiodine ablation and hypertension presented to the ED with a “sore throat.” The patient reported she had a sensation that something was choking her and preventing her from being able to swallow comfortably. She stated that she was able to tolerate liquids without difficulty, but swallowing solids was challenging. She reported that this issue had been progressing for at least several weeks, but she was unsure of the exact timeline. She had not had any changes to her weight and denied shortness of breath, voice changes, or drooling. She denied any new medications or any history of medication or food allergies. In fact, she had run out of all her medications three months prior including amlodipine 10 mg and levothyroxine 137 micrograms (mcg).

The patient’s vital signs were as follows: temperature, 97.2 °F (36.2 °C); heart rate, 68 beats per minute; blood pressure,130/69 millimeters of mercury; respiration, 16 breaths per minute; and oxygen saturation, 100% on room air. Physical examination revealed a generally well-appearing woman in no apparent distress. She controlled her secretions and phonated appropriately. The oropharyngeal exam revealed soft palate edema, which limited view of the posterior oropharynx. The tongue was not edematous. Neck exam revealed no palpable goiter, lymphadenopathy, or masses. Cranial nerve examination of nerves II–XII revealed no deficits. There was no stridor. The remainder of the patient’s examination did not reveal any abnormalities.

To adequately visualize the posterior oropharynx and vallecula, fiberoptic laryngoscopy was performed. The uvula, epiglottis, arytenoids, and vocal ligaments were without overt edema. Computed tomography of soft tissue of the neck with intravenous contrast confirmed isolated soft palatal edema and mild uvular edema without additional involvement (Image). No masses were appreciated.

Thyroid-stimulating hormone was markedly elevated at 123.337 milliunits/liter (mU/L) (reference range 0.35–4.0 mU/L) and free thyroxine (T4) was below normal limits at 0.3 nanograms/deciliter (ng/dL) (0.8–2.0 ng/dL). The patient’s soft tissue edema was attributed to palatal myxedema deposition. In consultation with the endocrinology service, levothyroxine was restarted at one-half her original dose for one week, after which she was to resume her full dose. The patient ultimately attended a follow-up endocrinology appointment with the consulting endocrinologist five weeks later, at which time she was documented to have full resolution of her symptoms.

## DISCUSSION

The differential diagnosis for a sore throat in the ED is broad, but the most common causes of sore throat are infectious such as pharyngitis or odontogenic infections. In older adults, dysphagia from primary esophageal pathology, neuromuscular disorder, or a mass lesion are of increased concern. When edema of the oropharynx is appreciated, angioedema is also a consideration whether allergic, medication-related, or hereditary.

*CPC-EM Capsule*

What do we already know about this clinical entity?

*A well-known feature of decompensated hypothyroidism is myxedema deposition.*

What makes this presentation of disease reportable?

*Isolated myxdema deposition in the soft palate has not been previously documented in the literature.*

What is the major learning point?

*Consider myxedema from decompensated hypothyroidism in patients presenting with oropharyngeal pain and/or swelling.*

How might this improve emergency medicine practice?

*This case of uncommon etiology of a common concern, sore throat, outlines diagnostics and treatment recommendations to prevent progression to airway compromise.*

Myxedema deposition into the oropharynx causing a presenting symptom of a sore throat is rare.^2^ To our knowledge, no other published cases that have described isolated soft palatal myxedema. Myxedema of the oropharynx can lead to respiratory failure and be acutely life-threatening, as detailed in several case reports, most typically published in the otolaryngology literature.^3^–^6^ For those at risk, myxedema deposition should be considered as part of the differential of sore throat, as early diagnosis and treatment will prevent progression and risk of airway compromise.

The prevalence of hypothyroidism in the United States is 0.3–3.7%. Primary hypothyroidism from autoimmune thyroiditis, radioiodine ablation, thyroidectomy, or radiation effects are most common. Older, female patients with a history of autoimmunity are at particular risk.^1^ Presenting symptoms of hypothyroidism are nonspecific, making this a challenging diagnosis. Features such as skin changes, cold intolerance, weight gain, and mood lability are unlikely to lead to an ED visit. While symptoms such as shortness of breath, eyelid edema, constipation or paresthesia may result in an ED visit, a diagnostic challenge may remain. Classic physical findings including coarse skin, hair loss, periorbital edema, voice hoarseness, hypotension, and altered mentation may be present when further decompensation occurs.

A less common physical exam finding in decompensated hypothyroidism is myxedema deposition, as in the case of our patient.^1^ Fiberoptic laryngoscopy in this case did not reveal evidence of posterior oropharyngeal edema; however, CT demonstrated edematous changes to the soft palate. If there is a suspicion for myxedema, thyroid testing to confirm clinical hypothyroidism should be sent. An elevated thyroid-stimulating hormone is highly suggestive of hypothyroidism. A low T4 distinguishes clinical hypothyroidism from subclinical hypothyroidism. Additional testing may be necessary if thyroid nodules are present or secondary hypothyroidism is suspected, or if the patient has cardiac comorbidities or evidence of myxedema coma.^1^

Hypothyroidism, in isolation, does not necessarily require hospitalization.^1^ In the context of oropharyngeal myxedema deposition, a thorough assessment of airway compromise is required. When subtle changes are appreciated on direct or fiberoptic visualization of the oropharynx or vallecula, consider a period of observation to monitor progression. In a patient with primary hypothyroidism who is appropriate for discharge, starting levothyroxine at 1.6 mcg/kilogram lean body mass is reasonable. In patients > 60 years of age or who have cardiac comorbidities, lower doses of 25–50 mcg/day can be initiated and up-titrated slowly over 6–8 weeks until goal dose is achieved. All patients require close endocrinologic follow-up for repeat lab testing within 6–8 weeks.^1^

## CONCLUSION

Although rare, myxedema deposition and decompensated hypothyroidism should be considered in those with a subacute presentation of a sore throat, dyspnea, or voice changes. Confirmatory testing with thyroid-stimulating hormone and thyroxine levels should be sent. Endocrinology consultation and disposition should be considered carefully. Early recognition could prevent progression to possible airway compromise.

## Figures and Tables

**Image:**
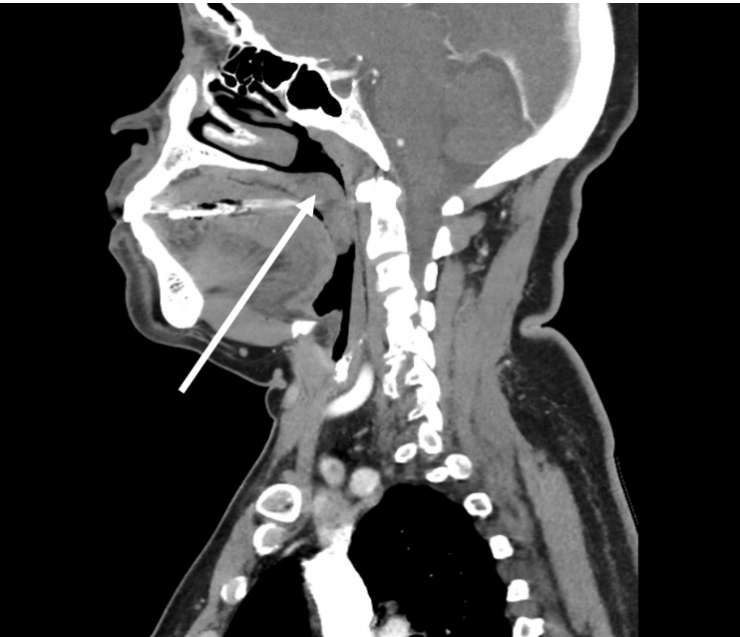
Soft palate swelling obliterating the space between the tongue and soft palate (arrow) in an older female patient who presented with a sore throat and was found to have myxedema deposition in the oropharynx.
